# Tuberculosis Preventive Treatment Scale-Up Among Antiretroviral Therapy Patients — 16 Countries Supported by the U.S. President’s Emergency Plan for AIDS Relief, 2017–2019

**DOI:** 10.15585/mmwr.mm6912a3

**Published:** 2020-03-27

**Authors:** Michael Melgar, Catherine Nichols, J. Sean Cavanaugh, Hannah L. Kirking, Diya Surie, Anand Date, Sevim Ahmedov, Susan Maloney, Rena Fukunaga, CDC Country Offices’ Tuberculosis/HIV Advisors, National Ministries and Departments of Health Tuberculosis Program Managers, CDC Country Offices’ Tuberculosis/HIV Advisors, Ho Thi Van Anh, Peter R. Kerndt, Deus Lukoye, Daniel Magesa, Talent Maphosa, Magdalene M. Mayer, Katlego Motlhaoleng, Bethrand Odume, Munyaradzi Pasipamire, Sarah Porter, Ahmed Saadani Hassani, James Simpungwe, Edwin Sithole, Herman Weyenga, National Ministries and Departments of Health Tuberculosis Program Managers, Gabriel Ekali, Clorata Gwanzura, Isaya Jelly, Muthoni Karanja, Patrick Lungu, Gugulethu Madonsela, Dorothy Maloboka, Ivan Manhiça, Do Thi Nhan, Stavia Turyahabwe, Emperor Ubochioma, Kgomotso Vilakazi Nhlapo, Thomas Webhale

**Affiliations:** ^1^Division of Global HIV and Tuberculosis, Center for Global Health, CDC; ^2^Epidemic Intelligence Service, CDC; ^3^Interagency Collaborative for Program Improvement, Washington, DC; ^4^Bureau for Global Health, Office of HIV/AIDS, USAID; ^5^Office of the Global AIDS Coordinator, Washington, DC.; CDC-Vietnam; CDC-Mozambique; CDC-Uganda; CDC-Tanzania; CDC-Zimbabwe; CDC-Cameroon; CDC-South Africa; CDC-Nigeria; CDC-Eswatini; CDC-South Africa; CDC-Zambia; CDC-Zambia; CDC-Namibia; CDC-Kenya; Ministère de la Santé Publique du Cameroun; Ministry of Health and Childcare, Zimbabwe; Ministry of Health, Community Development, Gender, Elderly and Children, Tanzania; Ministry of Health, Kenya; Ministry of Health, Zambia; Ministry of Health, Eswatini; Ministry of Health and Social Services, Namibia; Ministério da Saúde de Moçambique; Ministry of Health, Vietnam; Ministry of Health, Uganda; Federal Ministry of Health, Nigeria; National Department of Health, South Africa; Ministry of Health, Community Development, Gender, Elderly and Children, Tanzania

Tuberculosis (TB) is the leading cause of death among persons living with human immunodeficiency virus (HIV) infection. In 2018, an estimated 251,000 persons living with HIV infection died from TB, accounting for one third of all HIV-related deaths and one sixth of all TB deaths (*1*). TB preventive treatment (TPT) is recommended by the World Health Organization (WHO) for persons living with HIV infection without active TB disease (i.e., adults with a negative clinical symptom screen for cough, fever, night sweats, or weight loss; and children with a negative clinical screen for cough, fever, contact with a person with TB, or poor weight gain) and either without* a tuberculin skin test result or with a known positive result (*2*). TPT decreases morbidity and mortality among persons living with HIV infection, independent of antiretroviral therapy (ART) (*3*); however, in 2017, fewer than 1 million of the estimated 21.3 million ART patients started TPT worldwide. Most patients receiving TPT were treated with 6 months of daily isoniazid (*1*,*4*). This report summarizes data on TB symptom screening and TPT initiation and completion among ART patients in 16 countries supported by the U.S. President’s Emergency Plan for AIDS^†^ Relief (PEPFAR) during April 1, 2017–March 31, 2019. During this period, these 16 countries accounted for approximately 90% of PEPFAR-supported ART patients. During April 1, 2017–September 30, 2018, TB symptom screening increased from 54% to 84%. Overall, nearly 2 million ART patients initiated TPT, and 60% completed treatment during October 1, 2017–March 31, 2019. Although TPT initiations increased substantially, completion among those who initiated TPT increased only from 55% to 66%. In addition to continuing gains in initiation, improving retention after initiation and identifying barriers to TPT completion are important to increase TPT scale-up and reduce global TB mortality.

On September 26, 2018, the United Nations General Assembly held the first high-level meeting on TB and committed to providing TPT to 30 million persons by 2022, and the Office of the U.S. Global AIDS Coordinator (OGAC) announced a goal to provide TPT to all 13.6 million ART patients supported by PEPFAR by 2021 (*5*). PEPFAR-supported programs provide semiannual reporting on TPT initiation and completion for performance monitoring and evaluation, and the reporting cycle follows the U.S. government’s fiscal year (October 1–September 30). This report summarizes TB symptom screening and TPT data from 16 countries during the four most recent 6-month reporting periods.

Population estimates of persons living with HIV infection were obtained from the Joint United Nations Programme on HIV/AIDS (UNAIDS) public database (*4*). Additional data were submitted by PEPFAR implementing partners to OGAC, including the following required indicators: 1) the total number of ART patients; 2) the number of ART patients who completed TB symptom screening; 3) the number of those who screened negative; 4) the number expected to complete a standard course of TPT^§^; and 5) the number of those who completed a standard course of TPT (i.e., completion of at least 6 months of daily isoniazid or completion of an alternative regimen). This report describes the changes in these indicators among three groups of ART patients. Group 1 was screened during April 1–September 30, 2017 (period 1) and expected to complete TPT during October 1, 2017–March 31, 2018 (period 2); group 2 was screened during October 1, 2017–March 31, 2018 (period 2) and expected to complete TPT during April 1–September 30, 2018 (period 3); and group 3 was screened during April 1–September 30, 2018 (period 3) and expected to complete TPT during October 1, 2018–March 31, 2019 (period 4).

Of 54 PEPFAR-supported countries, 23 were required to submit annual PEPFAR country operational plans in fiscal years 2018 and 2019 (*6*) and were considered for inclusion in this analysis. Countries were excluded if they did not report data on both the number of ART patients expected to complete a course of TPT and the number who completed treatment during October 1, 2017–March 31, 2019 (periods 2–4). The percentage change in TPT completion per 100,000 ART patients from period 2 to period 4 was calculated for all countries except Cameroon and Zimbabwe because of small numbers of patients completing TPT in these countries. Overall, 16 countries, which account for 88% of PEPFAR-supported ART patients worldwide, reported sufficient data for TPT indicators and were included in this analysis. 

During April 1, 2017–September 30, 2018 (periods 1–3) across the 16 countries, the number of ART patients increased 11% (Figure 1). The number of these patients screened increased 71%, and the proportion of ART patients who underwent TB symptom screening increased from 54% to 84%; the number who screened negative increased 89%. During the entire study period, reported TB symptom screening among ART patients by country ranged from 35% (Namibia, period 1) to 108% (Vietnam, period 3) (Table). TB symptom screening results were missing for >10% of ART patients screened during one or more reporting periods in the Democratic Republic of the Congo (DRC), Ethiopia, Namibia, Uganda, Vietnam, and Zimbabwe.

**FIGURE 1:**
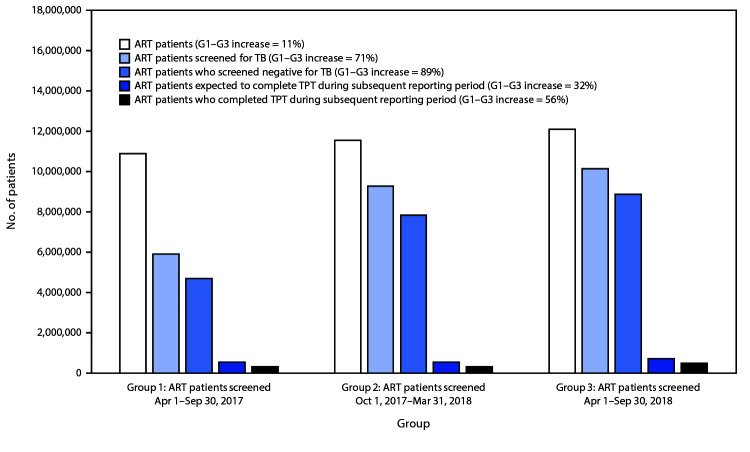
Tuberculosis (TB) screening and TB preventive treatment (TPT) indicators*^,^**^†^****^,^****^§^** for persons living with human immunodeficiency virus (HIV) infection receiving antiretroviral therapy (ART patients) — 16 PEPFAR-supported countries,**^¶^** 2017–2019 **Abbreviations:** G = group; PEPFAR = U.S. President’s Emergency Plan for AIDS Relief. * Number of ART patients reports a snapshot at the end of the reporting period, accounting for net gains (e.g., new HIV diagnoses) and losses (e.g., deaths and patients lost to follow-up). ^†^ The South African National Department of Health (NDOH) receives PEPFAR support for health system strengthening and other non–patient care activities. NDOH annually reports ART patients in its care but does not report TPT initiation or completion. Therefore, ART patients reported by NDOH were subtracted from South Africa totals in period 1 (779,313) and in period 3 (900,463) to include data reported only by South African partners that received PEPFAR support for delivering health care services. ^§^ Number of ART patients expected to complete TPT approximates number of TPT initiations. If the patient is prescribed 6 months of daily isoniazid, the patient is expected to complete treatment during the reporting period subsequent to that of initiation. Otherwise, if prescribed a shorter rifamycin-based course, the patient might be expected to complete treatment during the initiation reporting period. ^¶^ Cameroon, Democratic Republic of the Congo, Eswatini, Ethiopia, Haiti, Kenya, Lesotho, Mozambique, Namibia, Nigeria, South Africa, Tanzania, Uganda, Vietnam, Zambia, and Zimbabwe.

**TABLE:** Tuberculosis (TB) symptom-screening and TB preventive treatment (TPT) outcomes for persons living with human immunodeficiency virus (HIV) infection receiving antiretroviral therapy (ART), by country — 16 countries supported by the U.S. President’s Emergency Plan for AIDS Relief (PEPFAR), 2017–2019

Country (estimated no. of persons living with HIV infection)	No. of persons living with HIV infection receiving ART through PEPFAR*	No. (%) of ART patients screened for TB	No. of ART patients screened negative for TB	No. expected to complete TPT^†^	No. (%) completed TPT
**Group 1: TB screening during period 1** ^§^ **; TPT completion during period 2** ^¶^					
Cameroon (510,000)	176,927	130,795 (74)	126,893	276	41 (15)
DRC (390,000)	65,385	57,938 (89)	55,302	26,402	17,157 (65)
Eswatini (210,000)	150,987	137,590 (91)	134,638	9,394	7,744 (82)
Ethiopia (610,000)	434,897	386,419 (89)	334,407**	14,297	11,490 (80)
Haiti (150,000)	91,845	54,530 (59)	51,662	7,827	3,864 (49)
Kenya (1,500,000)	1,041,326	995,264 (96)	915,577	130,535	105,550 (81)
Lesotho (320,000)	151,799	136,522 (90)	132,230	5,298	765 (14)
Mozambique (2,100,000)	995,547	680,901 (68)	633,787	87,753	12,160 (14)
Namibia (200,000)	165,965	58,318 (35)	57,501	5,944	5,017 (84)
Nigeria (3,100,000)	772,510	660,501 (86)	606,726	45,093	34,840 (77)
South Africa (7,200,000)	3,256,407^††^	—^§§^	—^§§^	121,484	52,958 (44)
Tanzania (1,500,000)	932,425	893,280 (96)	880,954	59,660	27,264 (46)
Uganda (1,300,000)	993,070	960,999 (97)	77,857**	17,609	12,454 (71)
Vietnam (250,000)	87,702	43,173 (49)	30,766**	3,743	3,221 (86)
Zambia (1,100,000)	745,127	382,872 (51)	373,386	10,866	7,582 (70)
Zimbabwe (1,300,000)	849,310	338,535 (40)	287,622**	198	144 (73)
**Subtotal, period 1**	**10,911,229**	**5,917,637 (54)**	**4,699,308**	**546,379**	**302,251 (55)**
**Group 2: TB screening during period 2^¶^; TPT completion during period 3^¶¶^**					
Cameroon	177,434	148,143 (83)	143,684	1,385	888 (64)
DRC	72,143	65,127 (90)	39,356**	14,714	8,426 (57)
Eswatini	169,272	163,736 (97)	158,348	10,538	7,929 (75)
Ethiopia	451,436	384,226 (85)	262,289**	16,715	13,448 (80)
Haiti	95,697	81,280 (85)	73,622	8,229	4,839 (59)
Kenya	1,066,579	1,022,624 (96)	951,497	98,271	73,462 (75)
Lesotho	190,569	154,029 (81)	149,491	2,926	380 (13)
Mozambique	1,077,726	696,643 (65)	673,895	94,832	21,124 (22)
Namibia	177,062	112,221 (63)	49,317**	5,660	5,276 (93)
Nigeria	799,718	746,588 (93)	697,815	61,817	46,547 (75)
South Africa	3,446,694	2,540,588 (74)	2,440,961	124,520	65,526 (53)
Tanzania	995,953	968,540 (97)	939,591	64,279	31,534 (49)
Uganda	1,031,846	999,492 (97)	185,494**	8,246	5,248 (64)
Vietnam	91,457	74,761 (82)	11,123**	10,601	7,364 (69)
Zambia	801,669	296,204 (37)	289,812	17,225	13,233 (77)
Zimbabwe	939,164	839,120 (89)	766,572	290	134 (46)
**Subtotal, period 2**	**11,584,419**	**9,293,322 (80)**	**7,832,867**	**540,248**	**305,358 (57)**
Group 3: TB screening during period 3^¶¶^; TPT completion during period 4***					
Cameroon	188,979	158,850 (84)	152,459	8,526	3,324 (39)
DRC	88,488	85,026 (96)	54,051**	12,743	9,169 (72)
Eswatini	175,912	167,276 (95)	164,285	12,069	9,281 (77)
Ethiopia	460,565	427,242 (93)	346,031**	13,976	11,239 (80)
Haiti	101,597	87,865 (86)	84,588	10,454	6,095 (58)
Kenya	1,084,100	1,104,679 (102)	981,037	58,452	52,792 (90)
Lesotho	218,493	177,416 (81)	173,572	5,462	1,839 (34)
Mozambique	1,107,749	824,247 (74)	798,227	83,897	23,022 (27)
Namibia	179,844	88,706 (49)	47,487**	10,425	9,388 (90)
Nigeria	807,094	604,596 (75)	575,959	152,528	120,787 (79)
South Africa	3,515,553^†††^	2,986,266 (85)	2,896,646	139,721	66,045 (47)
Tanzania	1,075,346	1,063,362 (99)	1,041,380	126,352	96,737 (77)
Uganda	1,120,271	1,067,117 (95)	409,084**	19,103	13,419 (70)
Vietnam	122,822	133,101 (108)	28,277**	3,782	3,430 (91)
Zambia	894,090	514,926 (58)	524,954	37,761	26,859 (71)
Zimbabwe	968,690	653,288 (67)	604,467	23,270	17,836 (77)
**Subtotal, period 3**	**12,109,593**	**10,143,963 (84)**	**8,882,504**	**718,521**	**471,262 (66)**
**Total, periods 1–3**	**34,605,241**	**25,354,922 (73)**	**21,414,679**	**1,805,148**	**1,078,871 (60)**

**Abbreviation:** DRC = Democratic Republic of the Congo.

* Snapshot at end of the reporting period accounts for net gains (e.g., new HIV diagnoses) and losses (e.g., deaths and patients lost to follow-up).

^†^ Number of ART patients expected to complete TPT approximates number of TPT initiations. If the patient is prescribed 6 months of daily isoniazid, the patient is expected to complete treatment during the reporting period subsequent to that of initiation. Otherwise, if prescribed a shorter rifamycin-based course, the patient might be expected to complete treatment during the initiation reporting period.

^§^ April 1–September 30, 2017.

^¶^ October 1, 2017–March 31, 2018.

** >10% of TB symptom-screening results were missing.

^††^ The South African National Department of Health (NDOH) receives PEPFAR support for health system strengthening and other non–patient care activities. NDOH annually reports ART patients in its care but does not report TPT initiation or completion. Therefore, ART patients reported by NDOH were subtracted from South Africa totals in period 1 (779,313) and in period 3 (900,463) to include data reported only by South African partners that received PEPFAR support for delivering health care services.

^§§^ Indicator not reported.

^¶¶^ April 1, 2018–September 30, 2018.

*** October 1, 2018–March 31, 2019.

^†††^ The number of ART patients in South Africa was adjusted in Period 3 to include only data from sites receiving investment for direct health care service delivery.

Overall, 1,805,148 ART patients initiated TPT, and 1,078,871 (60%) completed treatment. During October 1, 2017–March 31, 2019 (periods 2–4), the number of ART patients who initiated and were expected to complete a course of TPT per period increased by 172,142 (32%), and the number who completed TPT per period increased by 169,011 (56%) (Figure 1). Although Nigeria and Tanzania accounted for <20% of all ART patients across the 16 countries, they accounted for 174,307 (101%) of the net increase in per-period TPT initiations and 155,420 (92%) of the net increase in per-period TPT completions (Table). Nigeria and Tanzania also experienced the largest increases in TPT completion rates per 100,000 ART patients (Figure 2). DRC, Ethiopia, Kenya, Uganda, and Vietnam reported fewer TPT completions per 100,000 ART patients in period 4 than in period 2.

**FIGURE 2:**
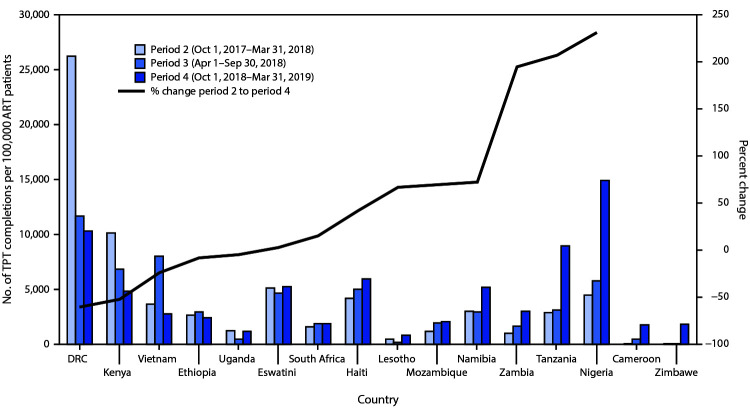
Number of ART patients (per 100,000 population) who completed tuberculosis preventive treatment (TPT) during three reporting periods,* and percentage change in TPT completion rate^†^— 16 countries supported by the U.S. President’s Emergency Plan for AIDS Relief (PEPFAR), October 2017–March 2019 **Abbreviations:** ART = antiretroviral therapy; DRC = Democratic Republic of the Congo. * The South African National Department of Health (NDOH) receives PEPFAR support for health system strengthening and other non–patient care activities. NDOH annually reports ART patients in its care but does not report TPT initiation or completion. Therefore, ART patients reported by NDOH were subtracted from South Africa totals in period 1 (779,313) and in period 3 (900,463) to include data reported only by South African partners that received PEPFAR support for delivering health care services. ^†^ Excluding Cameroon and Zimbabwe because of small numbers.

Although TPT initiations increased substantially during October 1, 2017–March 31, 2019 (periods 2–4), completion percentage among those who initiated TPT increased only from 55% to 66% (Table). In Tanzania, however, the percentage who completed TPT increased from 46% to 77%. Although TPT completion percentages increased substantially in Cameroon, Lesotho, and Mozambique, their completion percentages during the most recent reporting period, (along with TPT completion percentage in South Africa) remained <50%.

## Discussion

These findings represent substantial progress from the last decade, when fewer than 100,000 ART patients initiated TPT annually worldwide (*1*). This progress is likely attributable to increased engagement and collaboration among national TB and HIV programs, resulting in intensified TPT training for ART providers, alignment of national policies with WHO recommendations (*2*), and PEPFAR-led integration of TPT into standard HIV care (*7*). Progress, however, has not been consistent across countries and reporting periods. Nigeria and Tanzania have led the way in TPT scale-up. Driving the overall trend, these two countries made the most progress during the most recent reporting period.

Although TPT initiations have increased, gains in completion have been more modest. Tanzania, where the PEPFAR program intensively trained ART clinics on isoniazid supply chain forecasting and ordering during 2018–2019, is the most notable exception. With completion still <50% in several countries, identifying supply chain and other barriers to TPT completion might improve patient retention after TPT initiation and facilitate scale-up.

In Kenya, TPT initiation and completion decreased from the second to the fourth reporting period. However, Kenya is one of few countries that has tracked TPT coverage; an estimated 80% of all ART patients at PEPFAR-supported sites had received TPT by 2017 (*8*). Therefore, decreased TPT initiation and completion likely reflect decreased TPT demand, with most TPT requirement now among ART patients newly enrolling in care. This might apply to other countries with TPT programs that predate the assessment period, including Ethiopia, South Africa, and Vietnam. To estimate the TPT-eligible population and to set appropriate targets, countries could consider estimating historical TPT coverage and recording future cumulative coverage.

The findings in this report are subject to at least four limitations. First, incomplete reporting of TB symptom-screening results, such as in Namibia, Uganda, and Vietnam, leads to underestimation of the population eligible for TPT. However, in Kenya and Vietnam, TB symptom screening exceeded 100% in the third reporting period, possibly because some ART patients were screened and counted more than once. Along with lack of historical TPT coverage data in some countries, this double-counting leads to overestimation of the TPT-eligible population. Prioritizing data quality reviews, ideally in partnership with PEPFAR implementing partner agencies and national ministries of health, could improve TPT data quality. Second, this analysis did not identify barriers to TPT initiation and completion. Isoniazid stockouts affect many PEPFAR-supported countries (*9*) and might have reduced TPT completion in South Africa during these reporting periods (*10*). Third, this analysis included only ART patients in PEPFAR-supported programs, which do not represent all clinics that provide ART and TPT. Finally, several countries, including Malawi, have begun TPT scale-up but only began reporting initiation and completion of TPT in the two most recent reporting periods and were therefore not included in this report.

Increases in TPT initiation and completion in some of these 16 PEPFAR-supported countries demonstrate encouraging progress toward TPT scale-up. Continued TPT expansion among persons living with HIV infection has the potential to save hundreds of thousands of lives every year. Reaching all eligible ART patients will require intensified efforts to identify and overcome barriers to TB screening and TPT initiation, as well as to completion of treatment, and to ensure data quality across PEPFAR-supported countries.

**Summary**

**What is already known about this topic?**

Tuberculosis preventive treatment (TPT) decreases morbidity and mortality among persons living with human immunodeficiency virus infection but remains underutilized. The U.S. President’s Emergency Plan for AIDS Relief (PEPFAR) has committed to providing TPT to all eligible persons receiving antiretroviral therapy (ART patients) by 2021.

**What is added by this report?**

During April 1, 2017–March 31, 2019, TPT implementation improved substantially across 16 countries accounting for approximately 90% of all PEPFAR-supported ART patients. TPT initiations per reporting period increased 32%, and TPT completions increased 56%.

**What are the implications for public health practice?**

TPT expansion could save hundreds of thousands of lives annually. This will require all PEPFAR-supported countries to estimate the TPT-eligible population, identify barriers to initiation and completion of TPT, and improve data monitoring and reporting.

## Footnotes

* Testing for latent TB infection by tuberculin skin test or interferon-gamma release assay is not a requirement for initiating TPT in persons living with HIV. In resource-limited settings, these tests of infection are rarely accessible.

† Acquired immunodeficiency syndrome.

§ Number of ART patients expected to complete TPT approximates number of TPT initiations. If the patient is prescribed 6 months of daily isoniazid, the patient is expected to complete treatment during the reporting period subsequent to that of initiation. Otherwise, if prescribed a shorter rifamycin-based course, the patient might be expected to complete treatment during the initiation reporting period.
