# *Erratum*: Vol. 69, No. 12

**DOI:** 10.15585/mmwr.mm6922a4

**Published:** 2020-06-05

In the report “Tuberculosis Preventive Treatment Scale-Up Among Antiretroviral Therapy Patients — 16 Countries Supported by the U.S. President’s Emergency Plan for AIDS Relief, 2017–2019,” on page 329, an author’s academic degree was listed incorrectly. The correct degree is Sevim Ahmedov, **MD**.

